# Introduction to the Special Issue: Policies for Inclusive Development in Africa

**DOI:** 10.1057/s41287-022-00561-x

**Published:** 2022-08-21

**Authors:** Marleen Dekker, Nicky Pouw

**Affiliations:** 1 African Studies Centre Leiden University, Wassenaarseweg 52, 2300 RB Leiden, The Netherlands; 2 Governance and Inclusive Development , University of Amsterdam, Amsterdam, The Netherlands

**Keywords:** Inclusive development, Africa, Employment, Social protection, Governance, Development policy

## Abstract

While there is increasing academic analysis and policy concern regarding growing inequality and the need for more inclusive development trajectories, it is equally important to advance our understanding of the pathways to attain more inclusive development in practice. This paper serves as the introduction to a special issue examining the empirical outcomes and processes of inclusive development policies in selected countries in Africa. The paper presents a policy implementation and assessment framework as a lens that connects the different case studies. The framework links general inclusive development strategies in employment, social protection and governance, to the participation and representation of the various stakeholders as well as the monetary and non-monetary transaction costs in accessing and/or implementing these programmes on the ground in different national and sub-national contexts. Based on the findings of the 9 case studies, the paper also advances policy directions and operational frameworks to attain more inclusive development in practice.

## Résumé

Bien qu’il existe une analyse académique croissante, et une préoccupation politique envers la montée des inégalités et la nécessite de trajectoires de développement plus inclusives, il est tout aussi important d’avancer notre compréhension des chemins qui permettent—dans la pratique—d’atteindre un développement plus inclusif. Cet article sert d’introduction à un numéro spécial examinant les résultats empiriques et les processus de développement inclusifs dans certains pays en Afrique. L’article présente un cadre d’implémentation et évaluation des politiques, tel que un prisme qui connecte les différents études de cas. Ce cadre relie des stratégies de développement générales (dans l’emploi, la protections sociale, et la gouvernance) à la participation et la représentation de différents partis intéressés, aussi qu’aux aux couts de transaction (monétaires et autres) dans l’accès à ces programmes et à leur implémentation sur le terrain, dans des contextes nationales et sous-nationales. Nous basant sur les résultats de ces 10 études de cas, l’article avance des directions de politique et des cadres opérationnels, ayant comme but la réalisation pratique d’un développement plus inclusif.

## Background and Scope: Fostering Inclusive Development in Africa

Many African economies recorded high and sustained economic growth between the early 2000s until the onset of the Covid-19 pandemic in 2020. The distribution of the economic opportunities and other benefits of this growth has however not been equal and has been concentrated in specific economic sectors, specific geographical areas and specific groups within countries (Dekker 2017). The developmental benefits of growth failed to trickle down and spread to other sectors, regions and groups. Large groups of poor and vulnerable people remained excluded from increased welfare (UNDP 2012; Dulani et al. 2013); social indicators picked up only modestly, with unemployment remaining high, while income and other inequalities widened (INCLUDE 2013). At the same time, it became increasingly recognised, both in academic and policy circles, that the failure to structurally tackle these inequalities and to include the poor and vulnerable in economic growth and development poses a risk for the long-term sustainability of this economic growth (IMF 2015) and is undermining social cohesion and political stability (Gupta et al. 2015; Dekker 2017; Reinders et al. 2019; Krieger and Meierieks 2019; UNDP 2012).

There is increasing evidence that during the COVID-19 pandemic, poverty and socio-economic inequalities have deepened across the world, including in Africa. However, the application of “shock-responsive measures” in response to COVID-19, also generated some success in terms of horizontal and vertical expansion of poverty reduction and social protection programmes (Devereux 2021). Some scholars, therefore, argue the pandemic is an opportunity for African governments to rethink their social policies fundamentally by making them more inclusive and rights based (e.g. Ebuenyi 2020; Devereux 2021).

This call to build back better, or build forward more inclusively, aligns with the emerging consensus before the onset of the COVID-19 pandemic, on the need for more inclusive development (see for example UNDP 2012; UNECA 2015; Brookings 2018; Osakwe and Moussa 2017; AU-EU 2017). A policy focus on inclusive development suggests a move away from policy agendas that focus on economic growth only to a broader policy framework aimed at wellbeing, redistribution and equality of opportunity and outcomes in income, as well as non-income dimensions of development: Inclusive development occurs when average achievements on income and non-income dimensions of wellbeing improve and inequalities in these achievements fall (Kanbur and Rauniyar 2010; INCLUDE 2013). Moreover, inclusive development is not only about inclusive achievements or outcomes. It also resonates in building inclusive processes such as in policy formulation and implementation, and the quality of institutions (INCLUDE 2013). This policy shift builds on academic debates and conceptual development, such as advanced by Gupta et al. (2015) in this journal, Makandawire (2001), Adesina (2007), Hickey et al. (2014), and Van Niekerk (2020).

Along with the development of inclusive development as a concept, it is equally important to advance our understanding of the pathways to attain more inclusive development. The literature discussing policy options for more inclusive development is emerging and takes one of two approaches, the first having a focus on poverty reduction mostly, while the other is focusing on the reduction of inequality. Deriving from the key characteristics of the global poor, the Worldbank (2016) for example recommends a number of generic strategies that national governments can implement to reduce poverty and promote shared prosperity. These include for example, early childhood development and nutrition interventions, universal access to basic social services such as health and education, cash transfers to poor families, rural infrastructure and progressive taxation. Current drivers of inequality similarly provide levers to guarantee a minimum level of livelihoods (Adesina 2007) and promote more inclusive development***.*** Here, the existing literature advances at least three policy areas where sound national or sub-national public policies and strategic leadership by development-oriented public and private actors can make a difference in addressing inequalities (INCLUDE 2013):

1. High levels of underemployment and vulnerable employment in Africa (Adesina 2007), call for broad-based efforts by the state and private sector to increase productivity and generate more employment opportunities, more specifically, productive employment with decent working conditions and sufficient and stable remuneration (Szirmai et al. 2013). As the majority of the SSA population, including a high proportion of the poor, live in rural areas, agriculture and agribusiness will remain the largest employment category for at least the next decade. Tapping into the latent demand of (distant) markets through high value agricultural or agro-industrial chains allows (small holder) farmers to raise productivity and profitability that promotes their welfare and wellbeing. Moreover, through backward and forward linkages in local value chains, such increases in productivity, welfare and wellbeing will not be restricted to individual farming households, but under certain conditions have the potential to spillover to other rural families through employment opportunities, either on the farm or further down the value chain.
2. Broad based inclusive transformation also requires building-in redistributive mechanisms in the economic system. Cash transfers and social protection are commonly highlighted as effective policy instruments, not only to cushion vulnerability and promote resilience but also enabling productive and social investments by recipient households as well as the spillovers in the local economy (Gassmann 2014; Adesina 2012).
3. Broad based inclusive societal transformation also requires inclusive governance to promote a level-playing field between stakeholders (Adesina 2007; INCLUDE 2013). Inclusive development promotes more attention to the (diversity of) people within the system (de Haan 2015; Pouw and de Bruijne 2015) and is about inclusive development processes; processes of change that include the voices and perspectives of those who (risk to be) left behind.

Empirically documenting how, *where* and *when* such policies lead to more inclusive development and *for whom* informs policy making, design and implementation, and advances our conceptual thinking about inclusive development processes. In the NWO-WOTRO research programme ‘Research on Inclusive Development in Sub Saharan Africa’ (RIDSSA)^1^ 17 international research consortia studied various interventions, programmes, processes and actors in these policy domains in selected African countries (Ghana, Uganda, Ethiopia, Rwanda, Kenya, Nigeria, and Tanzania). The research consortia varied in disciplinary and stakeholder composition and employed different methods of data collection and types of analysis that straddled inclusive development outcomes and processes.

These empirical studies culminated in the nine cases contributing to this special issue. The cases cover the three broad policy domains of (1) employment in agricultural and industrial value chain development by local entrepreneurs, through multinational businesses or inclusive business models; (2) social protection policies such as social health insurance and cash transfers; and (3) the representation of indigenous ethnic groups and informal sector workers.

Grounded in these empirical case studies that consider inclusive development policies in practice, the papers in this special issue overwhelmingly demonstrate that ‘just’ investing in these policy domains is not a magic bullet to achieve more inclusive development. More specifically, as a collective, the studies contribute to the global debate in two distinct ways. First, the studies document how differences in opportunities lead to diverging outcomes. Pre-existing exclusionary mechanisms remain (partially) in place and these require extra efforts to be overcome. Second, the studies identify context-specific factors and transaction costs that curtail the opportunities of the poor to benefit from development processes, even if these appear inclusive in set-up and design. These factors and transaction costs point to policy options to further promote more equal opportunities and outcomes in inclusive development processes by shedding light on the required intermediate steps.

The remainder of this Introductory article is organized as follows. Section two presents a policy implementation and assessment framework as the lens that connects the different case studies. The framework connects the general inclusive development strategies to the participation and representation of the various stakeholders (inclusive development as a process) and the transaction costs in accessing and or implementing these programmes on the ground in different national and sub-national contexts. This is followed by an overview of the interventions and actors in the case studies and summary of the main findings in section three. How these findings translate into policy directions and operational frameworks is central to section four, after which section five concludes.

## Towards a Policy Analysis Framework

**Fig. 1:**
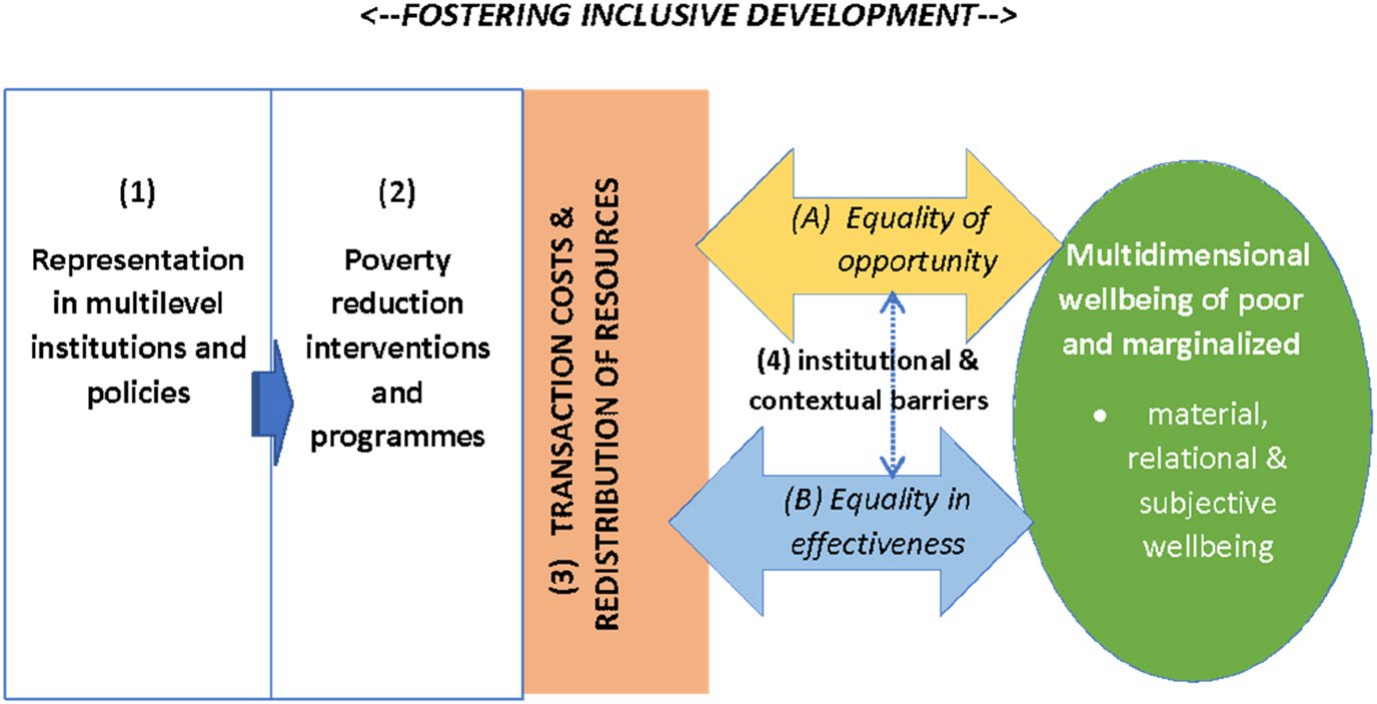
Conceptual framework: understanding inclusive development policy. Source: Authors

This section presents a policy analysis framework to understand inclusive development from a multi-dimensional wellbeing perspective of marginalized groups and development actors (see Fig. 1). It adds a policy and implementation perspective to the socio-economic elements of the conceptual inclusive development framework proposed by Gupta et al. (2015, EJDR SI on ‘Inclusive Development’).

Gupta et al. advanced three premises for implementation of inclusive development, namely: “(i) developing relevant epistemic communities, communities of practice and social movements, (ii) transforming governance into interactive governance to enable empowerment and (iii) adopting appropriate governance instruments” (pp. 549–550). The case studies in this SI build on these premises, by adding empirical scrutiny on the kind of strategic actors and interactive governance arrangements that can deliver inclusive development within a particular context and where there are gaps. We also add the specific importance of targeting social-economic and political mechanisms that could enhance redistribution, instead of focusing on the ‘level’ of economic performance alone. In doing so, the cases show that a clear focus on the poor and marginalized, the multi-dimensionality of wellbeing, and the heterogeneity of transaction costs to different groups of poor and marginalized, which are of paramount importance to finding points of entry for designing appropriate policy instruments and programmes.

Therefore, this framework includes the importance of:

1. the representation of the marginalized at multiple institutional and policy levels
2. how their priorities and needs are conceptualized and operationalized within poverty reduction policies and programmes, and
3. the transaction costs and redistributive resources related to access to and reaping the benefits of the interventions, programmes and processes, acknowledging that in particular contexts, inclusion may also be adverse (see also Hickey 2015). Transaction costs (Williamson 1979) vary from being more overt (i.e. visible and known to other actors and institutions) to ‘hidden’ (i.e. invisible and unknown). Moreover, transaction costs in general may be monetary (e.g. transportation) or non-monetary (e.g. waiting times; access to institutions) in nature. If development actors and institutions are unaware of these transaction costs, in particular the case of insurmountable costs (caused by level of frequency, idiosyncrasy and uncertainty faced by the very poor and marginalized or the stakeholders/development actors involved) the governance of such interventions and programmes may fail to effectively address poverty and function inclusively. Here we argue that to understand inclusive development it is essential not to study the interventions and related transaction costs in silo but in interaction with the
4. institutional, spatial and infrastructural context in which they are implemented (see Fig. 1).

Thus, transaction costs are inter-connected to an institutional and infrastructure context that potentially (re)produces exclusionary mechanisms, skewed outcomes or blockages, at macro- meso- and micro level of the society and the economy, preventing so-called trickle down or spillover effects in society and economy. As a result, the principle of equality of opportunity, that is often at the core of inclusive development policies, i.e. creating more opportunities, doesn’t automatically translate into equal use, outcomes and benefits (effectiveness (World Bank 2018)). As such, the conceptual framework endeavors to make a more policy oriented conceptual contribution to the inclusive development literature, which to date has been mainly theoretical in orientation.

It should be noted that this framework does not elaborate on the environmental sustainability dimension of the inclusive development framework as postulated by Gupta et al. (2015). The case studies in this SI did not contribute to empirical insights on the human-nature dimension, although we do recognize this being an important aspect of inclusive development.

## Overview of the Case Studies and Their Findings

The case studies in this special issue cover different analytical angles to and data on inclusive development processes, policies and interventions, including the perspectives from beneficiaries of programmes and interventions, the implementing and enabling actors (NGOs, private sector and government) as well as the institutional environment. An overview of the cases, their geographical locations, thematic foci and methods of data collection and analysis is presented in Table 1.

**Table 1:** Overview of case studies in this Special Issue

Author		Country		Intervention/actor	Process/outcome	Type of data	Analytical perspective and level of analysis
Agricultural and industrial value chains							
Muriitihi and Mariara		Kenya		Modern avocado value chains	Outcome/process	Panel survey data on avocado farmers and national policy documents	Micro-econometrics of small holder farmers
Van Paassen et al.		Ghana		Partnerships in cocoa, soybean and cassava value chains	Process/outcome	In-depth interviews key stakeholders Innovation Platforms and PPPs and focus group discussions with smallholder farmers	Micro-analysis of perspectives on partnership arrangements
Kebede et al.		Ethiopia		Inclusive business models in sesame, malt-barley and vegetable value chains	Outcome/process	Survey data on small holder farmers and focus group discussions	Micro-econometrics small holder farmers
Ezeoha et al.		Nigeria		Multi-National Organisations in dairy, beverage and cement industries	Process	National policy documents and MNC accounts	Multinational organisations
Social protection							
Van Reisen et al.		Uganda		Cash transfers and psycho social support	Outcome	Survey data on trauma relief and/or psycho-social support programme participants in a quasi-experimental design	Micro-econometrics of programme participants and non-participants
Gassmann et al.		Uganda		SAGE cash transfers and geographical	outcome	Focus group discussions and national policy documents and evaluations	Spatial inequalities in programme opportunities from an economic perspective
Pouw and Bender		Kenya Ghana		Social health insurance and conditional cash-transfers	Outcome/process	Interaction analysis of secondary data In-depth interviews key stakeholders in social protection Focus groups with beneficiaries and non-beneficiaries	Methodological reflections on combining micro-econometrics, community based assessments and political economy analysis
Representation and inclusive governance							
Goodwin	Rwanda		Decentralisation, pro-poor programming and accountability		Process/outcome	In-depth interviews and focus group discussions combined with national policy documents and evaluations	Socio-legal analysis of the perspectives of marginalized group
Kaag et al.	Ghana		Representation of informal sector workers		Process	Participant observation and in-depth interviews	Ethnography of membership organisations and their members

Each paper specifically addresses:

(i) A deeper analysis of policies, programmes, interventions and processes
(ii) The transaction or hidden costs to access opportunities and benefit from them, especially from the perspective of the ones who are, or risk to be, left behind, or, the unintended consequences resulting from existing policies and programmes.^2^
(iii) What could be done in the context of the specific policy and action instruments to reduce the hidden costs and promote more equity, and
(iv) How this is related to the specific regulatory, socio-economic, infrastructural and political context in which the study is set and the specific population groups and actors concerned.

An overview of these findings is presented in Table 2.

**Table 2:** Overview of Findings from the case studies

Author	Country	Intervention	Transaction costs	Hidden costs	Unintended consequences	Recommendations
Gassmann et al.	Uganda	SAGE cash transfers	The effect of access to transportation, access to telecommunications, and access to credit on engagement in agricultural wage labour, agricultural production, and non-farm trade	Unequal economic environment, local economic structures	Strengthening existing inequalities	Cash transfers should be accompanied by additional investment to level the playing field in benefitting from the transfers, else existing inequalities are widened
Van Paassen et al.	Ghana	Partnerships in food and cash crop value chains	Access to markets, storage processing and input services	Land, labour, organisation	Green revolution and free market logics overriding empowerment and social welfare logics in partnerships	Sharecropping overcomes access issues in cash crop value chain, organisation is important in the food value chain
Pouw and Bender	Kenya and Ghana	Social health insurance and conditional cash-transfers	Access to cash collection points, SHI renewal fee, picture on ID card, travel costs, access to referral mechanisms	Waiting times at health clinics, costs of medication, laboratory, and supplies (gloves, needles etc.) before being treated, negotiation costs due to misinformation about the free healthcare policy at local health centers, access to complaint and referral committees	Strengthening existing inequalities, e.g. when county/district budget allocations are made on the basis of political constituencies instead of where most poor and vulnerable people live; within communities envy and conflict when 'non-deserving poor' are included and 'deserving poor' are not	Improve synergies between different social protection policies and programmes
Ezaoa et al.	Nigeria	MNCs and employment creation and ID	Constrained institutional environment and unstable policies	Uncertainty and unequal competitiveness	Uncertainty leads to capital export and limited investment in local value chains	Stable policy environment to promote capital retention and provide incentives to MNCs to connect to local value chains to create added-value locally
Goodwin	Rwanda	Pro-poor programmes	Time constraints to attend meetings organised by local governments	Feelings of disconnect in understanding redistributive decisions and representation	Upward accountability results in a lack of downward accountability for some groups	Governance style of local government (redistributive decisions and redress)
Muriithi and Mariara	Kenya	Avocado value chains	Organisational membership to access training, input and markets	Ownership of land and time constraints make it more difficult for women to participate in farmers groups	Reaching ‘low hanging fruits’, unequal gender effects	Promote ownership of productive avocado varieties by women
Kaag et al.	Ghana	Representation of informal sector workers and collaboration between organisations (locally and internationally)	Competing political interests and agendas	Partisan logics create frictions between the informal workers themselves and those within the organisations in the informal economy that (cl)aim to represent them, and between these local organisations and their transnational partner organisations	Conflict and misrepresentation of informal sector workers	Unraveling of frictions as point of departure for identifying feasible and collaborative solutions based on a shared agenda
Kebede et al.	Ethiopia	Inclusive business models	Production and marketing standards too high, perceived risks of participation	Pre/intervention conditions (assets, crops, location of residence, acces to markets and finance), risk aversion, self exclusion	Participation in formal structures may lead to erosion of informal safety nets	Recognizing heterogeneity of farmers (initial conditions) and considering alternative supplementary measures to include the marginalized and excluded
Van Reisen et al.	Uganda	Cash transfers and psycho social support	Emotional costs, self exclusion	Trauma reduces the potential positive effect of cash transfers	Reaching only the ‘better-off’ and more capable	Coupling of trauma relief to social support programmes to enhance effectiveness

## Agricultural or Industrial Value Chain Development and the Generation of Productive Employment

The value chain studies in this SI consider the development of productive employment opportunities at the level of the value chain actors (MNCs, innovation platforms and strategic partnerships) as well their impact on and or (non) participation of smallholder farmers.

Ezeoha et al. (*this issue*) look at this from the perspectives of Multinational Corporations in the dairy, beverage and cement value chains that mainly produce for the local market in Nigeria, the largest economy on the continent. Specifically they study the level of integration of multinational organisations in local value chains (to identify backward and forward linkages that promote employment in the local vs the global economy) and their dividend policies that result in capital retention or export. Their review of the national policy environment documents a constrained institutional environment and high levels of uncertainty for firms. Local as well as foreign MNCs respond to this uncertainty by limiting their connection to local value chains and repatriating profits to preserve corporate value. The authors argue that policies that are tailored towards stabilizing the business environment will protect investments from risk of expropriation, and incentivize MNCs’ participation in the local value chains.

Van Paassen et al. (*this issue*) document the experiences with multi-actor partnerships for inclusive development in public private partnerships and innovation platforms in the export (cocoa) and food value chains (soy beans and cassava) in Ghana. They take a step back to explore local understandings of ‘inclusive development’ finding different interpretations and associations across stakeholders. Guided by institutional theory they assess how different stakeholders make use of different logics to frame their inclusive development initiatives, ranging from ‘green revolution’ and ‘free-market’ logics in the export sector, to ‘pro-poor’ solidarity logics in the smallholder farmer sector. These different framings have implications for partnership designs.

Muriithi and Kabubo-Mariara (*this issue*) study the opportunities of smallholder farmers to participate in the high value avocado export market in Kenya, with a specific focus on female farmers. In their analysis they highlight the intermediation of production marketing organisations (PMOs) in access to these remunerative markets and document that female farmers are less likely to be part of such organisations. Policy efforts should focus on supporting women farmers to enhance their participation in PMOs. Improving access to high-yielding avocado varieties, low cost agricultural credit and building capacity in orchard management would enhance women’s group participation, contracting and marketing.

In their contribution on Ethiopia, Kebede et al. (*this issue*) consider the demographic, asset-related and institutional determinants of inclusion and exclusion of small holder farmers in three distinct agricultural value chains (sesame, vegetables and barley) and specifically focus on the related income inequalities and value chain governance. Their analysis illustrates various perspectives on inclusion and exclusion, including self-imposed exclusion, and demonstrates how existing inequalities may deepen due to value chain participation. They call for additional policy measures that acknowledge the heterogeneity of the small holder farmers and support those who currently fail to benefit from the new opportunities provided.

## Social Protection

Gassman et al. (*this issue*) study spatial differences in making use of opportunities for productive investments and local economic spillovers, specifically considering the Senior Citizens Grant programme in Uganda. Based on a qualitative study design they find that the programme has unintentionally reinforced pre-existing social-economic inequalities across space; remoteness is found to correlate with underservicing and underperformance of social protection.

Van Reisen et al. (*this issue*) study mental health, or more specifically trauma, as a mitigating variable to use cash transfers for productive and social investments to promote resilience in Northern Uganda. Based on a quasi-experimental study design they find that providing trauma counseling contributes to social-economic resilience similar to cash/in-kind transfers. The failure to incorporate trauma counseling undermines the effectiveness of social protection programmes in conflict-affected areas.

Turning to Kenya and Ghana, Pouw and Bender (*this issue*) zoom in on social health insurance and conditional cash-transfers and specifically reflect on the use of mixed methods data collection and analyses to get at the more intricate (local) political economy of programme implementation and access. Based on a mixed methodology study design they show how little synergy is achieved between different social protection instruments, how material, relational and subjective wellbeing effects play out at household and community level, and how different social protection policies and instruments underdeliver (separately and jointly) to the very poor and poor, due to programmatic design failure and institutional and governance bias, underfunding and disconnects.

## Representation and Inclusive Governance

Although elements of representation and participation feature in the contributions on productive employment and social protection as well, two contributions in this SI specifically focus on the process of inclusive development. Kaag et al. (*this issue*) document the frictions that may arise when considering the possibilities to represent the constituents of informal sector workers organisations in Ghana. These frictions are found to be a source of information about the heterogeneity of political interests and agendas of different workers organisations and other stakeholders in their strive for ‘inclusive development’.

Goodwin (*this issue*) analyses the feelings of in- and exclusion of the Twa in decentralisation and anti-poverty programmes in Rwanda. From the perspective of the marginalized group, the Twa, she documents time constraints in attending local government meetings and a lack of understanding regarding both distributive decisions as well as representation at local level, resulting in feelings of exclusion in otherwise pro-poor programmes. Coupled with the incentives of local government officials to be accountable to the central government, Goodwin recommends paying more attention to downward accountability in the implementation of pro-poor programmes.

The contributions all illustrate that the implementation of inclusive development policies and programmes is messy and that both beneficiaries (f.e. Goodwin; Pouw and Bender; Reissen et al. *this issue*), and implementers (Ezoah et al.; Kaag et al.; *this issue*) are confronted with transaction and hidden costs (in time, risk, customary rules, asset ownership, incentive structures, mobility costs, etc.) to participate in or promote more inclusive outcomes and processes. Smallholder farmers f.e. may need to have access to productive resources (land, labour, high yielding varieties, etc.) in order to benefit from new opportunities provided by new value chains (Muriithi and Kububo-Mariara; Kebede et al. *this issue*).

The contributions substantiate how and why inclusive development can be challenged due to insurmountable thresholds to access and/or provide public goods and services, private sector markets and social and political institutions and services, to the extent that exclusion may be a deliberate choice for some as well (Kebede et al., *this issue*). Moreover, these thresholds play out at different levels of scale. These can be observed at (1) the national level, as documented by the constrained national policy environment in Nigeria by Ezoah et al. (2) at regional level, such as found in the spatial inequalities in spillover effects from social protection found in Uganda by Gassmann et al. (3) at local level as the spatial differences in the political economy of social protection implementation in Kenya described by Pouw and Bender, and in Rwanda by Goodwin and (4) at value chain level, as described by Muriithi and Kububo-Mariara and Kebede et al., or at community level as shown by Pouw and Bender.

The contributions also illustrate the challenges in effectively including poor people’s needs and priorities when establishing strategic collaborations (Van Paassen, *this issue*), strengthening informal economic activities and organization (Kaag et al., *this issue*), and providing productive employment (Ezaoh et al.; Kebede et al., *this issue*) and social protection tailor-made to different groups of poor (Gassmann et al.; Goodwin; Van Reisen et al., *this issue*) across the SSA countries studied. Taking the perspective of the implementation of policies and programmes, the cases document what can be done to make development more inclusive (the ‘extra mile’ to leave no one behind) and under what conditions (i.e. instrumental and contextual features) a policy, intervention, investment or action can be (more) bottom-up and functionally inclusive.

Finally, the case studies illustrate a number of conceptual theoretical and methodological lessons on how to do comprehensive research on inclusive development, as a process and outcome. These lessons take us back to the scholarly debates on ‘Inclusive Development’ versus ‘Inclusive Growth’, and the epistemological distinction between the two that requires further clarification in connection to the methodological approaches pursued. Where the realm of policies and instruments related to Inclusive Growth are primarily focused on market interventions and resolving market barriers and constraints, those related to Inclusive Development seem to take a broader array of policies and instruments and intervention domains into consideration, including multi-dimensional human wellbeing, social and ecological sustainability, and voice and empowerment (Gupta et al. 2015). As such, the latter approach is more cognizant of embedded power inequities in institutions and infrastructures, the entangled agency of people in the economic system also fulfilling labour tasks and services at home and in communities, and applies more often a mixed methodology approach including qualitative research geared at unraveling these and the complex trade-offs involved in situations where (painful) economic trade-offs are made.

## Lessons for Inclusive Development Policies

Before considering inclusive development policies that can generate a transformation of the observed status quo of structural inequalities and exclusionary mechanisms in the system in the above cases, what speaks from all the contributions is that ‘inclusion’ per se is not always favorable. Adverse inclusion in development programmes and interventions is, unfortunately, a standing practice meaning that intended beneficiaries trade-off different wellbeing aspects as well as wellbeing over time. For example, if jobs are created, but with unfavorable health or social conditions, or a microcredit programme that increases the debt and stress burden. Moreover, adverse exclusion can also occur as a side effect, due to unequal starting positions and lack of key resources (e.g. land, transport) that condition access (Kebede et al., *this issue*, Muriithi and Kabubo-Mariara, *this issue*), participation and the generation of complementary effects on local economies (Gassmann et al., *this issue;* Ezoha et al., *this issue*). Interestingly, some contributions in this SI posit that intended beneficiaries may also deliberately choose/decide not to be included in the process/intervention, for fear of adverse inclusion (Kebede et al.; Van Paassen, *this issue*).

Besides a critical eye on ‘inclusion’, the framing and subsequent communication about and implementation of ‘inclusive policies’ needs to be more unifying and strengthened by political commitment at national and decentralised levels (Pouw and Bender, *this issue)*. Although decentralisation has brought national politics in many African countries closer to citizens, this is not a guarantee for more inclusive visions or practices if upward and downward accountability are not balanced in favor of the latter (Goodwin, *this issue*). This requires more public debate and the opening-up of deliberative spaces to ensure representation of the marginalized, and possibilities for negotiating access and redistribution of resources. Standardized indicators of ‘the deserving poor’ can obfuscate those who are in real need of support or more equalized opportunities. The emphasis on economic policies over social policies in the ‘age of globalization’ in Africa (Shivji 2006) has led to a relative neglect of social policies (Muchie and Gumede 2017; Gumede 2018). It has also pointed out the need to self-develop the associated accountability mechanisms instead of accepting these as donor-imposed. More complementarity between economic and social interventions is needed (Gassmann et al., *this issue;* Van Reisen et al. *this issue*)*.*

Moreover, in the language of ‘inclusive development’, by framing intended beneficiaries as a homogenous group of dependents, development actors and institutions tend to overlook their uniqueness, knowledge and agency. This constitutes a missed opportunity for learning and better targeted interventions that could help in preventing negative unintended consequences for particular groups. All case studies in this SI call for a more emancipatory approach by establishing a level-playing field at multiple governance levels where mutual learning can be facilitated and inclusive instruments can be co-created for different participant groups and through different collaborations and forms of social learning (e.g. innovation platforms and PPPs; Van Paassen, *this issue*) to overcome competing interests (Kaag et al., *this issue*).

Collaborative action between stakeholders in development, should focus on shared concerns and flexibility in approaches, in order to overcome accustomed methods, competing interests, power contestations and diverging incentives. Diagnostic-type of approaches could be helpful in aligning different stakeholders to find context-specific understandings of the inclusive policies pursued in the process, and a more flexible range of solutions combining different strategic actors and instruments on a case-by-case basis.

The cases make clear that ‘the extra mile’ to positively and sustainably include the most marginalized requires stakeholders to view poverty, marginalization and exclusion as a social failure, and not only an economic problem. Every social problem plays out in three dimensions: a material (goods, resources, services, physical environment), relational (access to networks, institutions, representation, power) and subjective (psychological, emotional, mental) dimension (Van Reisen et al., *this issue*). The design of inclusive development policies and programmes, such as social protection in the form of cash/in-kind transfers and social insurances but also inclusive business models, need to take this multi-dimensionality into account because inequalities experienced within the different dimensions can have interlocking effects. Negative impacts in the social domain (e.g. ineffective alignment of formal and informal institutions; favoritism; conflictual interests) can run counter to material gains (e.g. number of economic activities in an area) in the long-run (Pouw and Bender, *this issue*). Social protection programmes targeting vulnerable populations, for example, should also pay attention to the psychological and psycho-social dimensions (Van Reisen et al., *this issue*). The feeling to be seen and ‘included’ in a justifiable manner is difficult to quantify, but may nevertheless be an important societal gain (Goodwin, *this issue*). To rely on market mechanisms alone for organizing access to resources, markets and public goods and services will not allow African economies to run the extra mile needed to put the last one first (Ezoha et al.; Gassmann et al.; Muriithi and Kabubo-Mariara, *this issue*).

Finally, cross-sector partnerships, involving public and private partners, provide a possible solution to revealing and overcoming hidden costs and conflicting agendas between different stakeholder groups (Van Paassen, *this issue*). Such meso-level strategies are best grounded in in-depth understanding of the diversified access to resources, institutions and networks, and decision making of marginalized groups. For example, solidifying informal sector workers’ access to markets and women’s participation in value chains, requires knowledge of their current positions and challenges. What hampers economic growth benefits to trickle down to them? How can such constraints be lifted? What would be needed to ease their challenges as informal sector workers first (as an intermediate step)? This implies a different approach (and starting-point) than the aim to formalize all informal sector workers. Inclusive development policies need to pursue a strong political representation of marginalized groups and open-up deliberative spaces in which their voices and differences can be mobilized, and self-development and empowerment can take shape.

## Conclusion and Lessons for Future Research

What is gained from the adoption of an ‘Inclusive Development Policy’ analytical framework (Fig. 1) is first of all, deeper insights into the heterogeneous nature and range of transaction costs faced by the poor and marginalized in accessing markets, social services and infrastructure and institutions. These transaction costs can be more overt or hidden, and can cover financial and social costs. How such costs play out differently for different sub-groups of poor and marginalized people, in terms of (in)equality of opportunity, requires further research. Second, economic and social policies and interventions have shown to have multidimensional wellbeing effects (see also Pouw 2020). However, how and why people trade-off different dimensions of wellbeing and such wellbeing over time (to capture intergenerational effects), is an area where more research is needed. Third, where cross-sector partnerships and a ‘learning by doing’ approach is found and advocated as an effective way forward by multiple studies, there remains a knowledge gap on how such collaborations could enhance social and political status and representation of poor and marginalized groups. Fourth, the findings point to a relative neglect of social policy development that is complementary to economic policies, as well as the design and implementation of bottom-up accountability mechanisms. This finding confirms earlier observations made by leading African scholars on the need to develop home-grown African social policy (Shivji 2006; Gumede 2018). Therefore, we recommend more African-led-research on home-grown pathways towards self-efficacy and social and political emancipation. Especially, a focus on the complementarity (or lack thereof) between social and economic policy frameworks, knowledge gaps are prevailing. This type of research would be particularly welcome as evidence-base in the current political debate of African countries on the ownership, professionalization and upscaling of national social protection policies and programmes. Fifth, and finally, mixed methodology approaches to studying the complex effects of inclusive development policies have led the researchers to novel insights on multi-level institutional barriers and constraints, multi-dimensional wellbeing effects missing components of social interventions to be really inclusive. Since these findings are largely based on cross-section data and ‘snapshots’ in time, the future research agenda should stimulate longer-term analysis and fund the required collection of time-series data and built-up of databases. The construction of such databases demands ‘patient’ and engaged institutions.

## Footnotes

1 The RIDSSA programme was conceptualised by INCLUDE, the Africa-Netherlands based Knowledge Platform on Inclusive Development Policies (see also INCLUDE 2013) and funded by the Netherlands Ministry of Foreign Affairs.

2 The case studies do not all address all of these dimensions, but each of the case studies speaks to at least one these transaction costs, hidden costs of unintended consequences.
